# Diabetic Endothelial Cell Glycogen Synthase Kinase 3β Activation Induces VCAM1 Ectodomain Shedding

**DOI:** 10.3390/ijms241814105

**Published:** 2023-09-14

**Authors:** Masuma Akter Brishti, Somasundaram Raghavan, Kennedy Lamar, Udai P. Singh, Daniel M. Collier, M. Dennis Leo

**Affiliations:** Department of Pharmaceutical Sciences, University of Tennessee Health Science Center, Memphis, TN 38163, USA; mbrisht1@uthsc.edu (M.A.B.);

**Keywords:** diabetic vascular disease, endothelial cells, soluble vascular cell adhesion molecule 1

## Abstract

Soluble cell adhesion molecules (sCAMs) are secreted ectodomain fragments of surface adhesion molecules, ICAM1 and VCAM1. sCAMs have diverse immune functions beyond their primary function, impacting immune cell recruitment and activation. Elevated sVCAM1 levels have been found to be associated with poor cardiovascular disease (CVD) outcomes, supporting VCAM1’s role as a potential diagnostic marker and therapeutic target. Inhibiting sVCAM1’s release or its interaction with immune cells could offer cardioprotection in conditions such as diabetes. Membrane-bound surface adhesion molecules are widely expressed in a wide variety of cell types with higher expression in endothelial cells (ECs). Still, the source of sCAMs in the circulation is not clear. Hypothesizing that endothelial cells (ECs) could be a potential source of sCAMs, this study investigated whether dysfunctional EC signaling mechanisms during diabetes cause VCAM1 ectodomain shedding. Our results from samples from an inducible diabetic mouse model revealed increased sVCAM1 plasma levels in diabetes. Protein analysis indicated upregulated VCAM1 expression and metalloproteases ADAM10 and ADAM17 in diabetic ECs. ADAMs are known for proteolytic cleavage of adhesion molecules, contributing to inflammation. GSK3β, implicated in EC VCAM1 expression, was found to be activated in diabetic ECs. GSK3β activation in control ECs increased ADAM10/17 and VCAM1. A GSK3β inhibitor reduced active GSK3β and VCAM1 ectodomain shedding. These findings suggest diabetic ECs with elevated GSK3β activity led to VCAM1 upregulation and ADAM10/17-mediated sVCAM1 shedding. This mechanism underscores the potential therapeutic role of GSK3β inhibition in reducing the levels of circulating sVCAM1. The complex roles of sCAMs extend well beyond CVD. Thus, unraveling the intricate involvement of sCAMs in the initiation and progression of vascular disease, particularly in diabetes, holds significant therapeutic potential.

## 1. Introduction

Diabetes mellitus is a chronic metabolic disorder characterized by hyperglycemia resulting from insulin resistance and/or impaired insulin secretion. The pathogenesis of diabetes involves complex immune and inflammatory processes that contribute to the development and progression of the disease [1,2]. Among the key players in these processes are soluble Intercellular Adhesion Molecule 1 (sICAM1) and soluble vascular cell adhesion molecule 1 (sVCAM1). This paper examines the role of insulin-resistance-triggered glycogen synthase kinase 3β (GSK3β) in endothelial cells as a potential source of circulating sVCAM1 in diabetic mice.

ICAM1 and VCAM1 are transmembrane glycoproteins expressed on the surface of various cell types, including endothelial cells and immune cells. They facilitate immune cell recruitment and adhesion during inflammation, which is crucial for the immune response. However, these adhesion molecules can undergo proteolytic cleavage during inflammatory conditions, releasing soluble forms into the bloodstream, referred to as sICAM1 and sVCAM1 [3,4]. Several studies have investigated the association between soluble CAM (sCAMs) levels and diabetes. Both sICAM1 and sVCAM1 have been implicated in the development of diabetic cardiovascular complications [5,6,7,8,9]. In type 2 diabetes (T2D), increased sICAM1 levels have been reported in the serum of diabetic patients compared to healthy individuals [6]. These elevated levels of sICAM1 are associated with insulin resistance, suggesting sICAM1’s potential as a biomarker for assessing diabetes risk and progression. sVCAM1 has particularly emerged as a biomarker in cardiovascular disease [7]. Elevated levels of sVCAM1 have been observed in diabetic patients, particularly those with poor glycemic control and complications [8]. Diabetes-related complications, such as retinopathy, nephropathy, and cardiovascular disease, are major contributors to morbidity and mortality in diabetic patients [10]. In diabetic retinopathy, increased sVCAM1 levels have been found to be associated with the severity of retinal vascular abnormalities and inflammation in mice and humans [9,11]. Moreover, sVCAM1 has been linked to diabetic nephropathy, with higher levels observed in patients with renal dysfunction [12]. Elevated levels of these soluble adhesion molecules are associated with endothelial dysfunction, atherosclerosis, and increased cardiovascular risk in diabetic individuals [13]. Thus, evidence suggests that sICAM1 and sVCAM1 may serve as indicators of diabetic complications. Given the role of sICAM1 and sVCAM1 in diabetic inflammation and complications, targeting these soluble adhesion molecules presents potential therapeutic implications. Inhibiting the expression or activity of sICAM1 and sVCAM1 could attenuate inflammatory responses and mitigate the progression of diabetic vascular disease. Preclinical studies have explored currently available and commonly used drugs and their effect on sICAM1 and sVCAM1 levels, demonstrating promising results in ameliorating diabetic vascular dysfunction [14,15]. Furthermore, emerging therapeutic strategies aim to directly target the interactions between sICAM1 and sVCAM1 with their receptors, preventing immune cell adhesion and inflammation in diabetes. Despite this, the source(s) for these increased circulating CAMs in diabetes or other conditions is not well defined. 

sICAM1 and sVCAM1 undergo proteolytic cleavage from their membrane-bound forms in a dynamic process significantly influenced by the actions of a family of enzymes called ‘A Disintegrin and Metalloproteases (ADAMs)’. Among these enzymes, ADAM10 and ADAM17 stand out as key players in the generation of soluble adhesion molecules [5,16,17,18]. The proteolytic activity of ADAM10 and ADAM17 is tightly regulated to maintain homeostasis and prevent excessive proteolysis. Several mechanisms control their activation and substrate recognition. The dysregulation of ADAM10 and ADAM17 activity has been implicated in various inflammatory diseases and pathological conditions. Altered cleavage of ICAM1 and VCAM1 by these metalloproteases may contribute to the pathogenesis of conditions such as atherosclerosis, rheumatoid arthritis, and cancer [16,17,18]. Understanding the regulation of these metalloproteases and their impact on soluble adhesion molecules may offer potential therapeutic targets for various inflammatory diseases. Here, we have uncovered that insulin resistance leads to the activation of GSK3β in mesenteric artery endothelial cells, which upregulates VCAM1 expression. GSK3β also independently activates ADAM10 and ADAM17 expression. Together, these metalloproteases cause the shedding of the VCAM1 ectodomain to increase extracellular levels of sVCAM1. A GSK3β inhibitor, tideglusib, was found to be effective in limiting activation of the enzyme and significantly reduced ectodomain shedding of VCAM1. Thus, this article highlights the source of sVCAM1 and identifies a potential checkpoint to prevent VCAM1 shedding that could improve cardiovascular function in diabetes.

## 2. Results

### 2.1. High-Fat Diet Combined with Low-Dose Streptozotocin Induces an Insulin-Resistant, T2D Phenotype in Mice

**Figure 1:**
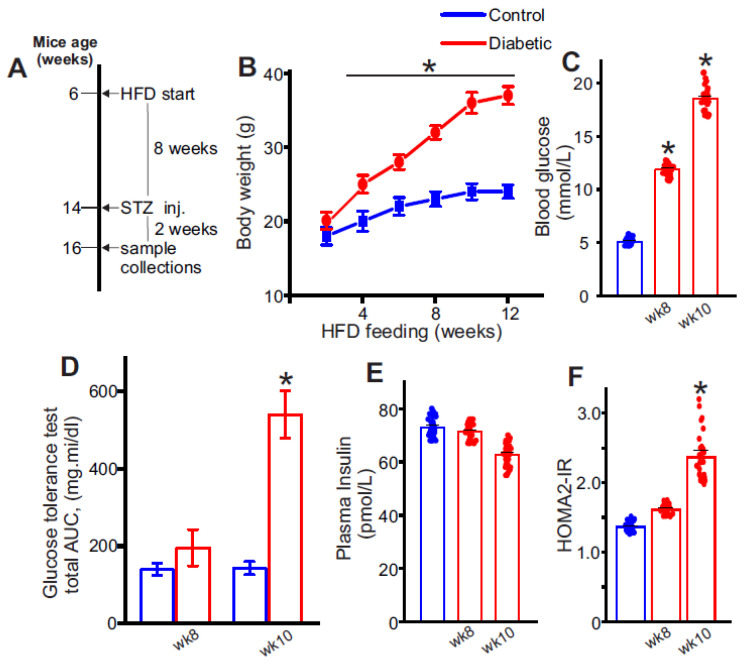
C57BL6/J mice on a high-fat diet with low dose-streptozotocin injections develop T2D. (**A**) Schematic of the HFD-STZ protocol. (**B**) Body weight (g) readings from week 2 of HFD (age: 6 weeks) until week 12. STZ was injected into mice after 8 weeks of HFD, and most mice were euthanized at 10 weeks. *n* = 6 for each data point. * *p* < 0.05 vs. nondiabetic control. (**C**) Fasting blood glucose (mmol/L) recordings from non-diabetic controls, week 8 of HFD prior to STZ and week 10 after STZ injections. *n* = 30 for each. * *p* < 0.05 vs. nondiabetic. (**D**) Oral glucose tolerance test in control and HFD mice before and after STZ at week 8 and 10 respectively. *n* = 6 for each. * *p* < 0.05 vs. nondiabetic. (**E**) Plasma insulin (pmol/L) in non-diabetic controls and HFD-STZ mice. *n* = 30 for each. (**F**) HOMA2-IR indices of non-diabetic, week 8 HFD and week 10 HFD-STZ mice calculated from values of (**C**,**E**). *n* = 30 for each. * *p* < 0.05 vs. nondiabetic controls.

Mice were subject to the HFD-STZ protocol to induce T2D as detailed in Figure 1A. Treatment mice showed a gradual increase in body weight from the start of the diet, with the animals after eight weeks of HFD weighing ~38 g (Figure 1B). The fasting blood glucose of the HFD-STZ mice also gradually increased over the same time (Figure 1C). For instance, 8 weeks after HD start, fasting blood glucose was ~13 mmol/L, compared with ~5.5 mmol/L in control mice, or ~2.36-fold higher (Figure 1C). After the STZ injections, body parameters were measured again, which indicated that fasting blood glucose was ~18 mmol/L or ~3.4-fold higher (Figure 1C). The glucose tolerance test (GTT) was performed as it was in our previous publication [19] and expressed as area under the curve (AUC) [20]. Results indicate that although GTT showed an upward trend after 8 weeks of HFD, it was after the STZ injections that glucose tolerance was significantly affected in the diabetic mice (Figure 1D). Next, the HOMA2-IR surrogate index was used to determine the level of insulin resistance in these mice. Fasting blood glucose (Figure 1C) and plasma insulin values (Figure 1E) were uploaded into the HOMA2-IR calculator described in the methods and HOMA2-IR indices were recorded. Results indicate that 8 weeks after the start of HFD, HOMA2-IR index was ~1.6 (Figure 1F). However, 2 weeks after STZ injections, the HOMA2-IR index was ~2.5 (Figure 1F). These data indicate that a high-fat diet combined with STZ leads to obesity, and insulin resistance in mice which resembles a T2D phenotype.

### 2.2. Plasma Soluble VCAM-1 (sVCAM1) Levels Increase with Insulin Resistance

**Figure 2:**
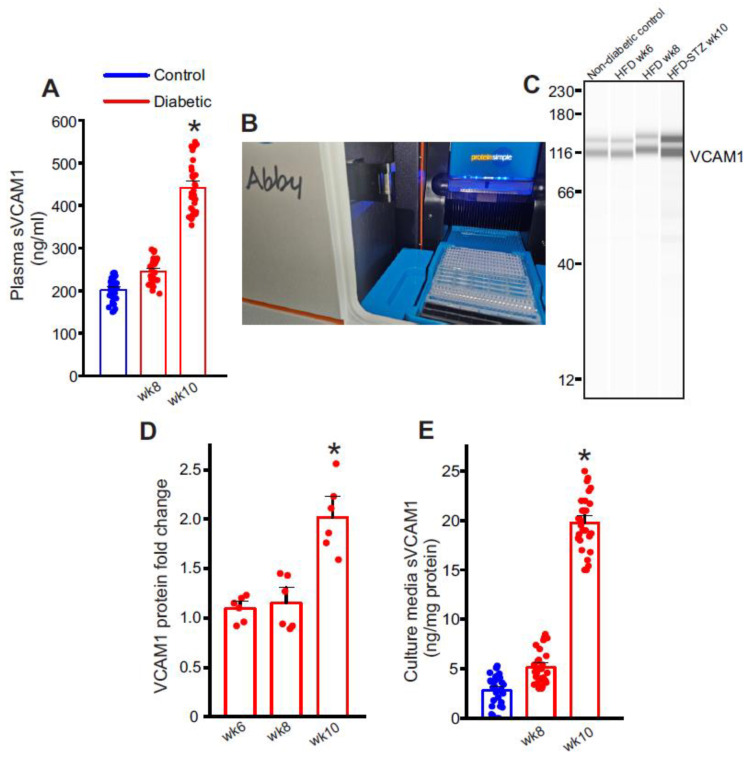
Circulating sVCAM1 levels increase after STZ injections and onset of insulin resistance. (**A**) Plasma sVCAM1 levels measured from nondiabetic and HFD mice before (week 8) and after STZ (week 10) injections. *n* = 30 for each. * *p* < 0.05 vs. nondiabetic. (**B**) Photograph of the Abby^TM^ (ProteinSimple) Simple Western instrument showing loaded lanes and capillary setup. (**C**) Representative full-length Simple Western of VCAM1 protein in isolated control and diabetic endothelial cells. (**D**) Mean data of VCAM1 protein expression in diabetic ECs. *N* = 6 each. * *p* < 0.05 vs. nondiabetic controls. (**E**) Mean data of sVCAM1 levels in culture media of ECs isolated from nondiabetic or diabetic mice. *n* = 30 each. * *p* < 0.05 vs. nondiabetic controls.

To verify if the occurrence of insulin resistance influences circulating sVCAM1 levels, plasma from control and diabetic mice was tested using an sVCAM1 ELISA kit. Results suggest that plasma sVCAM1 levels were slightly elevated in pre-insulin-resistant mice, approximately after 8 weeks of HFD alone (Figure 2A) but was not significantly different from control mice. In contrast, there was a significant increase in plasma sVCAM1 in the mice after STZ injections that became insulin resistant (Figure 2A). These data suggest that the occurrence of insulin resistance triggers an increase in circulating levels of sVCAM1.

### 2.3. Endothelial Cells as a Possible Source of Plasma sVCAM1 in Diabetic Animals

Adhesion molecules like VCAM1 are known to be widely expressed in endothelial cells. Hence, we postulated that sVCAM1 shedding occurs from endothelial cells during T2D. To analyze this, mesenteric artery endothelial cells were isolated as described previously and primary cultured for 8–10 days. sVCAM1 levels in the cell culture media were sampled on day 8 of culture after which the cells were lyzed for protein analysis. Results from ELISA of the culture supernatant revealed that sVCAM1 levels were significantly greater in diabetic cells isolated after STZ (week 10) injections than controls or those isolated before STZ injections (week 8, Figure 2E). Next, the expression of VCAM1 in endothelial cells was analyzed using the ProteinSimple Abby^TM^ capillary electrophoresis Western system (Figure 2B). Results indicated that there was significantly more upregulation of VCAM1 expression in diabetic endothelial cells after STZ injection than before (Figure 2D). These data suggest that insulin resistance might have a critical role in inducing both the expression of VCAM1 and the shedding of sVCAM1 from endothelial cells.

### 2.4. Diabetic Endothelial Cells Have Upregulated ADAM10 and ADAM17 Expression

**Figure 3:**
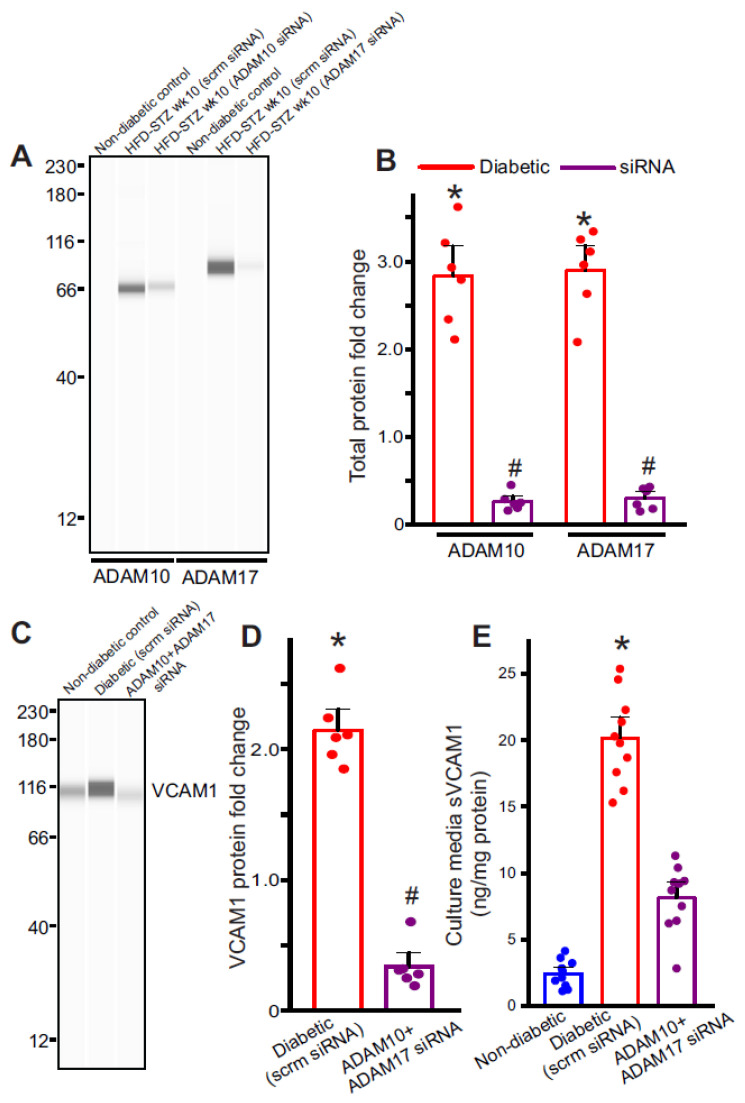
Metalloproteases ADAM10 and ADAM17 are involved in VCAM1 ectodomain shedding. (**A**) Representative full-length Simple Western of ADAM10 and ADAM17 proteins in isolated control and diabetic endothelial cells and after siRNA transfection. (**B**) Mean data of ADAM10 and ADAM17 protein fold change in week 10 HFD-STZ diabetic ECs and protein knockdown. *n* = 6 each. * *p* < 0.05 vs. nondiabetic controls, # *p* < 0.05 vs. diabetic. (**C**) Representative full-length Simple Western of VCAM1 protein from ECs of non-diabetic, diabetic transfected with scrambled or ADAM10 and 17 siRNA. (**D**) Mean data of VCAM1 protein fold change after ADAM10 or ADAM17 protein knockdown. *n* = 6 each. * *p* < 0.05 vs. non-diabetic controls, # *p* < 0.05 vs. diabetic scrambled siRNA. (**E**) Mean data of sVCAM1 levels in culture media of ECs isolated from scrambled siRNA or ADAM10 and ADAM17 siRNA transfected ECs. *n* = 8–9 each. * *p* < 0.05 vs. scrambled siRNA transfected.

Next, to determine the likely signaling causing VCAM1 shedding in diabetic endothelial cells, we examined the expression of two metalloproteases, ADAM10 and ADAM17, which had been previously suggested to cause VCAM1 shedding. In isolated cells from diabetic mesenteric arteries, the expression of ADAM10 and ADAM17 was upregulated (Figure 3A,B) which coincided with the increase in plasma and extracellular sVCAM1 levels. Next, to verify whether the upregulated ADAM10/17 also caused VCAM1 shedding, we silenced ADAM10 or 17 expression in diabetic ECs using specific siRNAs and analyzed protein expression. Results indicate that ADAM10 and ADAM17 siRNAs decreased expression of either protein ~70% each compared to diabetic ECs transfected with scrambled siRNA (scrm) (Figure 3A,B). When ADAM10 and 17 were silenced, there was also a decrease in cellular VCAM1 expression (Figure 3C,D) and sVCAM1 in the extracellular media (Figure 3E). These results suggest that the upregulation of ADAM10 or 17 in diabetic endothelial cells likely causes an increase in sVCAM1 shedding. Since we had also observed an increase in VCAM1 protein expression, we next investigated the possible mechanisms involved. 

### 2.5. Diabetic Endothelial Cell GSK3β Regulates the Expression of ADAM10/17 and VCAM1

**Figure 4:**
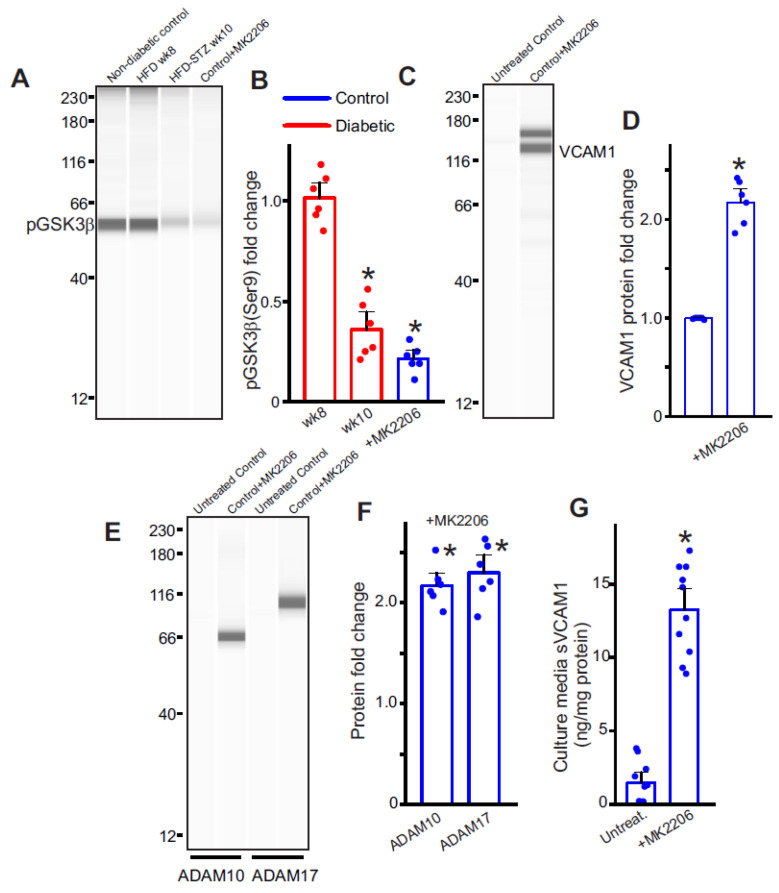
GSK3β activation induces sVCAM1 shedding. (**A**) Representative full-length Simple Western of pGSK3β (Ser9) protein in isolated control and diabetic endothelial cells. (**B**) Mean data. *n* = 6 each. * *p* < 0.05 vs. nondiabetic or untreated controls. (**C**) Representative full-length Simple Western of VCAM1 in controls with/without MK2206. (**D**) Mean data of VCAM1 protein expression in control ECs with/without MK2206. *n* = 6 each. * *p* < 0.05 vs. untreated controls. (**E**) Representative full-length Simple Western of ADAM10 or ADAM17 proteins in control ECs with/without MK2206. (**F**) Mean data. *n* = 6 each. * *p* < 0.05 vs. untreated controls. (**G**) Mean data of sVCAM1 levels in culture media of ECs treated with MK2206. *n* = 10 each. * *p* < 0.05 vs. untreated.

Insulin resistance is associated with dysfunctional insulin receptor signaling, affecting the activity of the downstream Akt kinase. Since basal Akt activity phosphorylates and deactivates GSK3β, we hypothesized that insulin resistance likely led to an activation of GSK3β, which induced ADAM10/17 and VCAM1 expression. To verify this, we first probed for levels of inactive GSK3β (pGSK3β-Ser9). Results indicate that pGSK3β levels rapidly fell after the STZ injections in HFD mice (Figure 4A,B), suggesting that insulin resistance was key to activating endothelial GSK3β. We then treated control endothelial cells with MK2206, a specific pan-Akt inhibitor, to inactivate Akt and thereby stimulate GSK3β activation. The results indicate that in the presence of the Akt inhibitor, pGSK3β levels significantly decreased (Figure 4A,B). In addition, when GSK3β was activated by the Akt inhibitor in ECs, the expression of ADAM10, 17, and VCAM1 also significantly increased (Figure 4C–F). In the presence of the Akt inhibitor, MK2206, there was also an increase in sVCAM1 present in the cell culture media (Figure 4G). These results suggest that GSK3β activation in endothelial cells is responsible for an upregulation of VCAM1 expression. GSK3β is also likely involved in ADAM10/17 upregulation, both of which cleave VCAM1 and cause its shedding. 

### 2.6. Selective Inhibition of GSK3β Downregulates Plasma sVCAM1 Levels

**Figure 5:**
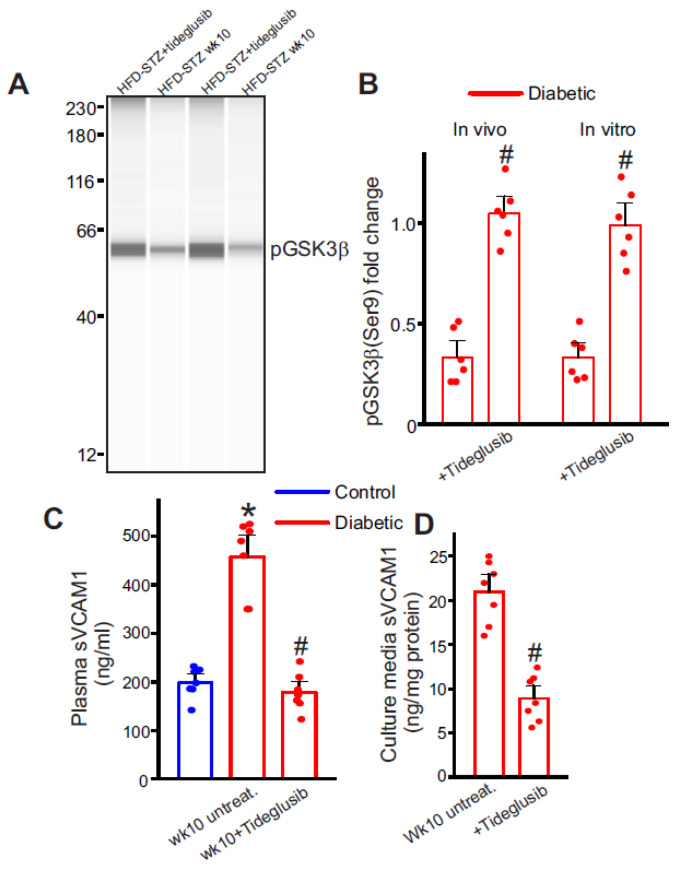
Inhibition of GSK3β activation reduces sVCAM1 shedding. (**A**) Representative full-length Simple Western of pGSK3β (Ser9) protein in isolated control and diabetic mesenteric artery samples and ECs. Lanes 1 and 2 are mesenteric artery lysates, while lanes 3 and 4 are EC lysates. (**B**) Mean data. *n* = 6 each. # *p* < 0.05 vs. respective untreated diabetic. (**C**) Plasma sVCAM1 levels measured from nondiabetic and HFD mice after STZ (week 10) injections with/without Tideglusib treatment. *n* = 6 each. * *p* < 0.05 vs. nondiabetic, # *p* < 0.05 vs. untreated diabetic. (**D**) Mean data of sVCAM1 levels in culture media of week 10 HFD-STZ mesenteric artery ECs treated with Tideglusib in vitro. *n* = 7 each. # *p* < 0.05 vs. week 10 HFD-STZ ECs untreated.

We then tested whether GSK3β inhibition was a viable option to decrease plasma sVCAM1 levels in diabetic animals. HFD mice that were injected with STZ were allowed a recuperation period of 3 days to allow the elimination of STZ. Tideglusib injections were commenced after this period for 2 weeks until mice were euthanized at the end of 10 weeks. As before, plasma samples and mesenteric arteries were collected at the end of the treatment period for analysis. ECs were also collected after STZ injections and were treated with tideglusib in vitro. For this, tideglusib (100 nM) was added to the EC culture along with fresh media every day. Results from ELISA show a significant drop in plasma sVCAM1 levels compared to untreated controls (Figure 5A). Protein analysis also showed a significant increase in pGSK3β levels in both whole mesenteric artery samples (Figure 5C,D) and cultured diabetic endothelial cells (Figure 5C,D). The sVCAM1 levels of the extracellular media were also significantly reduced compared to untreated diabetic cells (Figure 5B). These data indicate that diabetic EC GSK3β inhibition can decrease the circulating levels of sVCAM1.

In summary, these results indicate that insulin resistance increases circulating levels of sVCAM1. The activation of GSK3β in ECs after insulin resistance upregulates VCAM1 expression, and VCAM1’s ectodomain is cleaved by the metalloproteases ADAM10 and ADAM17, which also show increased expression in diabetic ECs. The inhibition of diabetic EC GSK3β can reduce levels of circulating sVCAM1 and could decrease cardiovascular disease risk and confer long-term cardiovascular protection. 

## 3. Discussion

The role of circulating soluble cell adhesion molecules (sCAMs) as indicators of cardiovascular disease risk has been extensively debated. However, the precise sources for these sCAMs have yet to be identified. Cell adhesion molecules are expressed in a wide variety of cells. Still, given their robust expression in endothelial cells (ECs) and the large surface area in the body that these cells occupy, it was reasonable to hypothesize endothelial cells as potential sources of sCAMs observed in the plasma during disease. This study examined whether dysfunctional ECs secrete sCAMs, the potential signaling mechanisms involved, and possible therapeutic targeting to decrease sCAM levels in diabetic mice. Our results collectively suggest that endothelial cells from insulin-resistant diabetic mice have upregulated GSK3β activity, which increases VCAM1 expression and the expression of metalloproteases ADAM10 and 17, leading to the cleavage of VCAM1 and the shedding of its extracellular domain into the plasma as sVCAM1.

ICAM1 and VCAM1 are transmembrane glycoproteins expressed on the surface of various cell types, including endothelial cells and immune cells. They facilitate immune cell recruitment and adhesion during inflammation, which is crucial for a satisfactory immune response [3,4]. However, these adhesion molecules can undergo proteolytic cleavage during inflammatory conditions, releasing soluble forms into the bloodstream, referred to as soluble ICAM1 (sICAM1) and soluble VCAM1 (sVCAM1) [3,4,5,6,7,8,9]. These soluble forms of CAMs are involved in diverse immune processes beyond their cell adhesion roles [5,6,7,8,9,21,22,23]. They participate in leukocyte migration, transendothelial migration, and immune cell activation, influencing the immune response’s overall outcome [3,21,23,24].

Cardiovascular disease (CVD) is a leading cause of mortality worldwide, necessitating the identification of reliable biomarkers for early diagnosis and prognosis. Several studies have investigated the association of sICAM1 and sVCAM1 with CVD, and their potential as diagnostic markers has been a subject of extensive debate [4,21,22,23,25,26,27,28,29,30]. Elevated levels of both sICAM1 and sVCAM1 have been reported in individuals with CVD, with sVCAM1 levels emerging as a particularly promising predictor of adverse outcomes in cardiovascular conditions [7,13]. Researchers have proposed using monoclonal antibodies or small molecule inhibitors to target sICAM1 and sVCAM1 to dampen excessive immune responses in inflammatory diseases [25,28]. Such therapeutic interventions could mitigate tissue damage and reduce disease severity in rheumatoid arthritis and inflammatory bowel disease. Moreover, the significance of sVCAM1 in cardiovascular disease suggests its potential as a therapeutic target for managing atherosclerosis and related complications [4,21,22,23,25,26,27,28,29,30]. The inhibition of sVCAM1 release or its interaction with immune cells could slow the progression of atherosclerosis and reduce the risk of cardiovascular events. Aside from their potential as diagnostic markers, these soluble CAMs have also garnered attention as potential therapeutic targets in various diseases, extending beyond CVD. Some research studies have investigated their involvement in cancer, indicating their possible relevance in a broader range of conditions [31,32,33]. This expanding knowledge highlights the importance of understanding the complex roles of these soluble adhesion molecules in various disease contexts. 

Results from our study show that plasma sVCAM1 levels increased after the onset of insulin resistance. We used the HFD-low dose STZ injections to induce insulin resistance in C57BL/6 mice. The C57BL/6 HFD-low dose STZ (HFD-STZ) model of type 2 diabetes (T2D) is an exceptional tool that closely imitates the polygenic nature of human disease [34,35,36,37,38,39,40,41]. We chose the inducible model to precisely time the occurrence of insulin resistance in our study mice. It is worth noting here that this model’s efficacy largely depends on the diet composition. Most studies employing this model (without STZ injections) utilize feed with 60% of its adjusted calories derived from fat. Despite this high-fat content, a considerable period of 9–11 weeks is still required to achieve the reliable induction of insulin resistance [42,43]. US diets are estimated to be 30–40% energy from fat [44,45,46]. In our study, we utilized the TD.88137 ‘Western diet’, which derives ~45% of its calorific value from milk fat. We also used an updated version of the HOMA-IR index calculator to estimate insulin resistance in our mice [47,48]. Our experimental mice typically achieved a HOMA2-IR index of ~2 or above, an accepted threshold for IR, after diet only for twelve weeks or more. Still, the administration of low-dose STZ injections at ~8 weeks of HFD reliably induced insulin resistance with calculated HOMA2-IR indices of >2. Our next goal was to determine the source of soluble VCAM1.

Endothelial dysfunction is commonly observed in diabetic vasculature [49,50]. However, the crosstalk between endothelial cells and the immune system is still unclear. Given their large surface area, we tested the hypothesis that ECs specifically from the mesenteric artery, are likely sources of circulating sVCAM1. In this study, extracellular sVCAM1 levels were increased in the media of cultured ECs from diabetic arteries. Interestingly, EC VCAM1 protein levels were also increased, suggesting that protein expression and cleavage are upregulated in diabetic ECs. 

ADAMs, a group of transmembrane matrix metalloproteases (MMPs), play a significant role in the proteolytic cleavage of adhesion molecules and chemokines [51]. In diabetic vasculature, the dysregulation of ADAMs has garnered significant interest due to their pivotal role in inflammation, cell adhesion, and extracellular matrix remodeling. ADAM10 and ADAM17 have been extensively studied in the context of diabetic vasculature. These metalloproteases are involved in the proteolytic cleavage of cell surface molecules, such as CX3CL1, VCAM-1, ICAM-1, and JAM-A, which play essential roles in endothelial cell function and leukocyte adhesion [5,16,17,18]. Dysregulated ADAM activity can increase sCAM ectodomain shedding, contributing to endothelial dysfunction and the recruitment of inflammatory cells in diabetic vasculature [52]. In diabetic retinopathy, the ADAM17-mediated cleavage of CX3CL1 and VCAM-1 in retinal endothelial cells has been associated with vascular leakage and retinal neovascularization [53]. Moreover, the ADAM10-mediated shedding of CX3CL1 has been linked to diabetic nephropathy, where increased levels of soluble CX3CL1 contribute to kidney injury and fibrosis [54]. In several CVDs, the ADAM10- and ADAM17-mediated cleavage of adhesion molecules has been implicated in atherosclerosis and vascular remodeling [55]. The elevated levels of sCAMs have been suggested to be important mediators of progressive endothelial dysfunction seen in the diabetic vasculature that is often refractive to commonly employed anti-diabetic treatment strategies. In our study, expectedly, we found an increase in the expression of ADAM10 and ADAM17 in diabetic ECs. Hence, it was highly likely that one or both enzymes mediate the ectodomain cleavage and shedding of EC VCAM1. 

We found that the critical instigator in EC VCAM1 expression and sVCAM1 shedding was GSK3β. Our data showed an increase in active GSK3β in ECs after the insulin resistance, which was associated with a concurrent increase in VCAM1 and ADAM10 and 17 expressions. Insulin acts via the insulin receptor to activate downstream Akt signaling [56,57,58,59]. Insulin resistance is associated with dysfunctional Akt signaling [56,57,58,59]. Phosphorylation of GSK3β at serine-9 is critical in regulating the activation of GSK3β in cells. Akt kinase downstream to the insulin receptor is one of several kinases that can deactivate GSK3β [56,57,58,59]. GSK3β activation was shown to increase EC VCAM1 expression after TNF-alpha treatment [60]. Hence, it is likely that activated GSK3β directly increased the expression of VCAM1 in our diabetic ECs. Similarly, while active GSK3β was upregulated in diabetic ECs, the activation of GSK3β in control ECs using an Akt inhibitor was followed by an increase in ADAM10 and 17 expressions. These results suggest that 1. basal Akt activity in ECs plays a major role in modulating GSK3β activation, and 2. ADAM10/17 upregulation was likely associated with the increase in GSK3β activation. Several other kinases are known to be activated in diabetes and it is possible that GSK3β is only one of the mechanisms that triggers ADAM10 and 17 upregulation. Finally, we tested the effect of a specific GSK3β inhibitor on EC sVCAM1 shedding. Tideglusib (NP-12, NP031112) is a selective and irreversible GSK3β inhibitor that was previously in clinical trials for Alzheimer’s disease and progressive supranuclear palsy [61,62,63]. Although withdrawn for lack of efficacy in these conditions, the drug has remained a vital investigational tool. Tideglusib was administered to diabetic mice three days after the completion of the STZ injections. Mice received the drug only with no other concurrent anti-diabetic therapy to help better identify the specific effects of GSK3β inhibition. One week of drug administration significantly decreased the whole mesenteric artery and EC GSK3β activation. There was also a significant drop in plasma and extracellular media sVCAM1 levels. These data suggest that arterial/EC GSK3β inhibition can significantly reduce the circulating levels of sVCAM1 and may offer long-term cardiovascular protection, especially when combined with other anti-diabetic drug therapies.

The interpretation of the upregulation of ADAM10 and ADAM17, particularly in ECs, is not straightforward due to its context-dependent implications. EC surface adhesion molecules, such as VCAM1, play a crucial role in mediating leukocyte attachment and infiltration. This suggests that VCAM1 ectodomain shedding by metalloproteases may help to regulate the local inflammatory response. However, in the context of diabetes, multiple factors, including the activation of GSK3β, can trigger a self-sustaining deleterious feedback loop, further enhancing the expression of these metalloproteases. Recent research has shown that EC ADAM17 can cleave the insulin receptor ectodomain, leading to cellular insulin resistance [64], which could exacerbate insulin resistance, with the initial upregulation of ADAM17 potentially leading to further GSK3β activation. Moreover, the role of circulating sVCAM1 and sICAM1 is likely more complex than anticipated. These circulating CAMs could induce the activation of circulating and resident immune cells, thus triggering an inflammatory response. Hence, the sheddase activity of ADAMs in the diabetic vasculature, along with the impact of released ectodomains into the circulation, needs renewed attention. 

## 4. Materials and Methods

### 4.1. Animal Usage

All experimental protocols were in accordance with institutional guidelines approved by the Institutional Animal Care and Use Committee, UTHSC. Male C57BL/6J were used for experiments, and diabetic mice were generated using the high-fat diet (HFD)-low dose streptozotocin (STZ, Sigma Aldrich, St. Louis, MO, USA) protocol described in our previous publication [19]. Briefly, 6 week old mice were started on HFD. Mice in the HFD group were fed with TD.88137 (Inotiv, Chicago, IL, USA, previously Envigo). Following eight weeks of HFD, mice were injected with low-dose STZ (40 mg/kg/d, IP, 4 doses) to induce insulin resistance, and were continued on HFD for another 2 weeks. Mice were euthanized before or after STZ injections as required. Blood glucose was assessed using AlphaTrak2 glucose test strips, and plasma insulin was evaluated using a mouse insulin ELISA kit (Crystal Chem, Elk Grove Village, IL, USA). The homeostasis model assessment-2 (HOMA2-IR) index was calculated using the calculator available on the Diabetes Trials Unit of the University of Oxford website (https://www.dtu.ox.ac.uk/homacalculator/; accessed: 1 June 2022) [47], as has been carried out previously [39]. Tideglusib (10 mg/kg/d, IP, for one week, Sigma Aldrich) was started 3 days after the treatment group’s final dose of STZ and continued until the end of the experimental period. Tideglusib was dissolved in 4% DMSO + corn oil for in vivo administration. 

### 4.2. Tissue Preparation

For the collection of arteries, mice were humanely euthanized with an overdose of isofluorane anesthesia followed by decapitation. Mesenteric arteries were collected and cleaned in ice-cold physiological saline solution (PSS) that contained (in mM) 112 NaCl, 6 KCl, 24 NaHCO_3_, 1.8 CaCl_2_, 1.2 MgSO_4_, 1.2 KH_2_PO_4_, and 10 glucose, gassed with 21% O_2_, 5% CO_2_, and 74% N_2_ to pH 7.4, and processed for downstream applications. 

### 4.3. Endothelial Cell (EC) Isolation and Culture

ECs were isolated following a standard protocol described in our previous publication with slight modifications [65]. EC basal media (Endothelial cell GM MV2, PromoCell, Heidelberg, Germany) containing 2 mg/mL collagenase type 1 (Worthington Biochemical, Lakewood, NJ, USA) were gently pushed into the isolated mesenteric arterial lumen using a tuberculin syringe and left to incubate for 30–40 min at 37 °C after which the cells were gently flushed out with EC basal media. The isolated cells were passed through a CD31 magnetic microbead containing separation column (Miltenyi Biotec, Gaithersburg, MD, USA) for positive selection of ECs. With the column placed in a magnetic stand, two washes of the column were performed with BSA containing phosphate-buffered saline (PBS) to flush out other cell types. Finally, the CD31 bead-bound cells were flushed into media containing EC basal media (PromoCell) and manufacturer-recommended supplements (PromoCell). The culture media were replaced on day 2 of culture and subsequently on days 4 and 7. Most cultures achieved confluency by day 7. After replenishment of media on day 7, the cultures remained undisturbed for 4 days, at the end of which the media were sampled for sVCAM1 levels and cells were lyzed for downstream analysis. For siRNA studies in diabetic ECs, a similar approach was followed, except that siRNAs were introduced 12 h prior to the media replacement on day 7. The cultures then remained undisturbed for 4 days and sVCAM1 levels were estimated at the end, similar to control cells, to measure the effect of siRNA-induced silencing of ADAM10 and 17 on sVCAM1 levels.

### 4.4. siRNA-Mediated Knockdown Studies

siRNAs directed to mouse ADAM10 and ADAM17 were purchased from ThermoFisher Scientific, Inc. Waltham, MA, USA. For silencing, 15 µg of each siRNA was incorporated into Lipofectamine 3000 reagent (ThermoFisher Scientific) and added to each well of primary diabetic ECs. Media were replaced 12 h after introduction of lipofectamine, as detailed in Section 4.3, and knockdown effect was measured after 4 days. Cells were then lysed, and proteins isolated for capillary electrophoresis blotting or other analysis. The siRNAs were not tested in control cells, as the expression levels of ADAM10 and 17 were too low to observe any measurable difference.

### 4.5. sVCAM1 ELISA

Soluble VCAM-1 levels in the plasma were assayed by using human-soluble VCAM-1 ELISA kit (ThermoFisher Scientific) as per manufacturer’s instructions. Briefly, for quantification of sVCAM-1, the plates were washed twice with 400 μg of wash buffer (PBS with 0.05% Tween 20) followed by 100 μL of Assay Buffer and 100 μL of diluted plasma and incubated in each plate well. Fifty micrograms of the provided biotin-conjugated anti-human sVCAM-1 was added to the well and incubated for 2 h at room temperature. Following three washes, 100 µL of substrate solution (tetramethylbenzidine) was added to each well and incubated for 10 min at room temperature. 1 M phosphoric acid was then added as a stop solution and the absorbance analyzed at 450 nm using an ELISA reader. Test readings were then plotted against a standard curve to determine sample concentrations.

### 4.6. Abby^TM^ Capillary Electrophoresis Immunoassay (Simple Western)

Mesenteric artery isolates and EC lysates were homogenized in ice-cold RIPA buffer (25 mM Tris pH 7.6, 150 mM NaCl, 1% Igepal CA-630, 1% sodium deoxycholate, 0.1% SDS) with protease inhibitor cocktail (Sigma Aldrich) using a mechanical homogenizer. The homogenate was centrifuged at 10,000 rpm for 5 min at 4 °C, and the supernatant was collected. Protein concentration was quantified with the Amido Black technique. Proteins were then resolved with capillary electrophoresis using the Abby^TM^ system (ProteinSimple, San Jose, CA, USA). An equal amount of protein was loaded into each well of the cartridge. The following primary antibodies were used: anti-VCAM1 (MAB6432, Biotechne, Minneapolis, MN, USA), anti-ADAM10 (14194, Cell Signaling Tech, Danvers, MA, USA), anti-ADAM17 (NBP1-77044, Novus Bio., Centennial, CO, USA), and anti-phospho-GSK3β (9323, Cell Signaling Tech). Following this, the anti-rabbit secondary antibody detection module (ProteinSimple) was used to detect the band of interest. Each individual capillary can be probed with a different primary antibody if needed, which allows simultaneous probing of multiple antibodies on one capillary cartridge. Protein expression was normalized to the total protein present in each lane, as conducted in prior publications [66,67,68]. For this, manufacturer-provided Replex reagent^TM^ (ProteinSimple) was used to strip the antibodies from the lanes after their detection, followed by use of the total protein detection module (ProteinSimple), which utilizes biotin labeling of all proteins. Bands were then analyzed using the built-in Compass^TM^ software v6.3.0 (ProteinSimple). The full lengths of capillary runs are shown for representative images. Total protein lanes of each main figure are shown in the Supplementary Figure S1. Band intensities of each lane were normalized to the total protein band intensities of the respective samples and expressed as fold change compared to control or non-diabetic samples.

### 4.7. Statistical Analysis

OriginLab v10 and GraphPad InStat v3.1 software were used for statistical analysis. Data are expressed as means ± SE. Student’s *t*-test, Mann–Whitney *U* test, and ANOVA with Bonferroni’s post hoc test for multiple group comparisons were used where appropriate. *p* < 0.05 was considered significant.

## 5. Conclusions

In conclusion, in this study we show that circulating sVCAM1 levels are increased in insulin-resistant HFD-fed mice. This increase was found to be due to an increase in the expression of the metalloproteases ADAM10 and ADAM17 in diabetic ECs, whose expressions were triggered by the activation of GSK3β. The inhibition of GSK3β in vitro and in vivo decreased VCAM1 ectodomain shedding and could possibly offer cardioprotection in diabetes.

## Data Availability

The data presented in this study are available in the article.

## Supplementary Materials

The following supporting information can be downloaded at: https://www.mdpi.com/article/10.3390/ijms241814105/s1.
