# Supplementary figures and images for: Diabetic Endothelial Cell Glycogen Synthase Kinase 3β Activation Induces VCAM1 Ectodomain Shedding
Source: Int J Mol Sci. 2023 Sep 14;24(18):14105. doi: 10.3390/ijms241814105 (PMC10531890; doi:10.3390/ijms241814105)

**Figure S1. Total protein lanes of each main figure (A-G).**

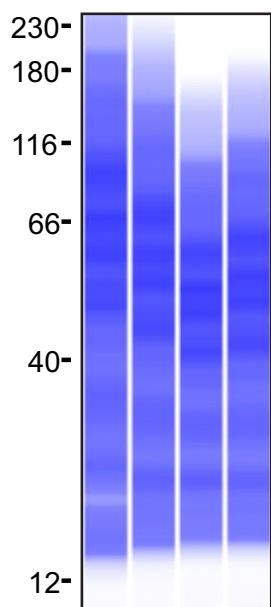

A

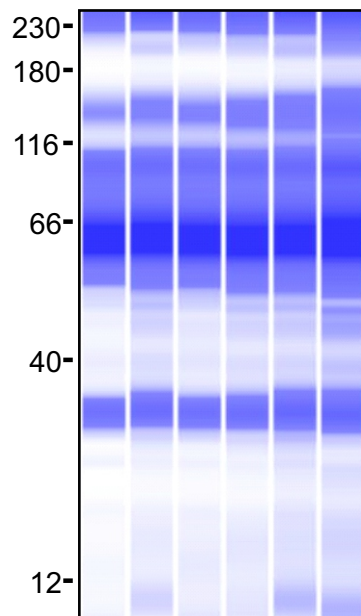

B

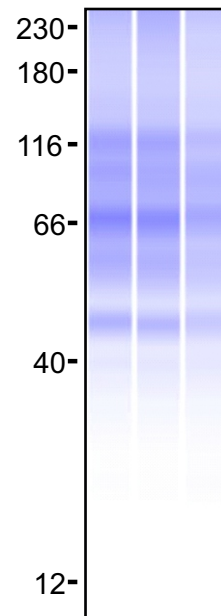

C

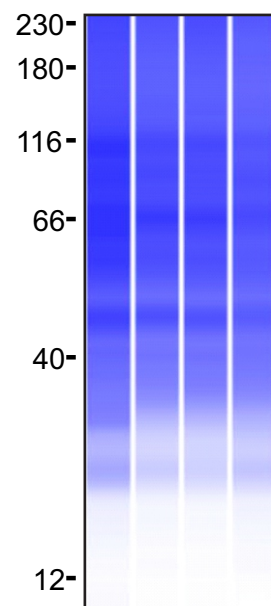

D

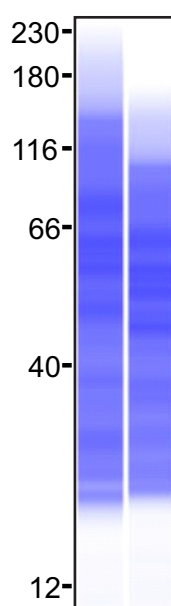

E

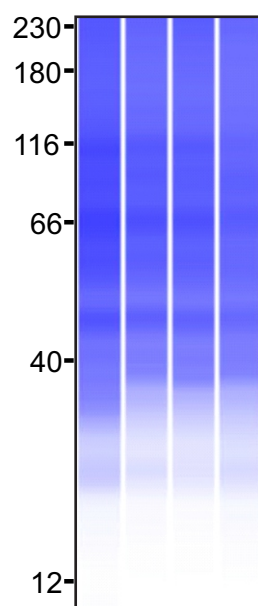

F

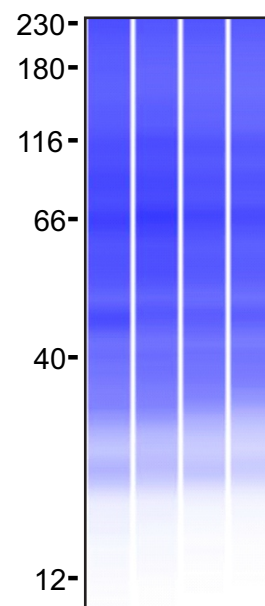

G

Supplement: Supplementary file 1 [file ijms-24-14105-s001.zip › ijms-2581156-supplementary.pdf]
